# Cardiovascular and Cerebrovascular Outcomes Risk Reduction Associated With Semaglutide vs Tirzepatide: Methodological Concerns

**DOI:** 10.1016/j.jacadv.2026.103402

**Published:** 2026-10-30

**Authors:** Jianghe Yan, Guangting Zeng

**Affiliations:** The First People's Hospital of Chenzhou, Xiangnan University, Chenzhou, China

We read with great interest the target trial emulation study by Azzam et al^1^ comparing cardiovascular and cerebrovascular outcomes between semaglutide and tirzepatide. However, several methodological aspects of this study merit further clarification.

First, there are some inconsistencies in the data. Two sensitivity analyses (Supplemental Tables 1 and 4 of Azzam et al^1^ study) report divergent risk differences and attenuation ratios for identical incident case endpoints. For example, the 1-year risk difference for incident atrial fibrillation in nondiabetic patients is 0.4% in Table 1 but only 0.1% in Table 4, with mismatched attenuation ratios. Supplemental Table 2 also contains an apparent computational error: the 3-year risk difference for acute coronary syndrome in nondiabetic patients is 0.09%, yet its 95% CI is listed as 0.10-0.10, where the point estimate falls outside the interval and both bounds are identical. Furthermore, it is worth noting that after propensity score matching was conducted within the time windows of 1 year and 3 years, an unexpected sample size pattern emerged: the number of matched patients in the type 2 diabetes cohort at 3 years (n = 95,614) was greater than that at 1 year (n = 77,178). These numerical discrepancies will reduce the transparency of the research results and require clarification.

Second, authors mention that causal estimand is per-protocol effect approximated via propensity scoring in target trial emulation flow diagram (Figure 1 of the Azzam et al^1^ study), yet all analyses follow an intention-to-treat framework: patients remain assigned to their initial treatment group regardless of subsequent medication switches, discontinuation, or treatment nonadherence. No inverse probability of censoring weighting, per-protocol restriction, or other methods to adjust for nonpersistence was applied. This mismatch between plan and implementation can lead to a blurring of causal explanations. From a methodological perspective, it is also necessary to clarify that propensity score addresses issue of baseline confounding, making the baseline characteristics of the 2 groups comparable and ensuring the exchangeability of conditions. On the other hand, per-protocol effect emphasizes the issue of compliance and ensures consistency. Therefore, the propensity score cannot achieve an approximate per-protocol effect. Furthermore, overly heterogeneous exposure definitions affect the consistency assumption. Given that different dose regimens confer distinct cardiometabolic benefits, this exposure misclassification dilutes effect estimates and limits clinical translatability.

In summary, although this study provides valuable exploratory real-world evidence, these methodological limitations warrant caution in interpreting tirzepatide’s superior cardiovascular protection. Clarification of the causal estimand and correction of data discrepancies would greatly strengthen the robustness of the findings.
